# Frightened of giants: fear responses to elephants approach that of predators

**DOI:** 10.1098/rsbl.2023.0202

**Published:** 2023-10-11

**Authors:** Robert J. Fletcher, Amanda O'Brien, Timothy F. Hall, Maggie Jones, Alex D. Potash, Laurence Kruger, Phumlile Simelane, Kim Roques, Ara Monadjem, Robert A. McCleery

**Affiliations:** ^1^ Department of Wildlife Ecology and Conservation, University of Florida, Gainesville, FL 32611, USA; ^2^ School of Animal, Plant and Environmental Sciences, University of the Witwatersrand, Johannesburg, South Africa; ^3^ Organization for Tropical Studies, Skukuza, South Africa; ^4^ All Out Africa, Savannah Research Centre, Mbuluzi Game Reserve, Lubombo, Eswatini; ^5^ Department of Biological Sciences, University of Eswatini, Kwaluseni, Eswatini; ^6^ Mammal Research Institute, Department of Zoology & Entomology, University of Pretoria, Pretoria, South Africa

**Keywords:** Africa, megafauna, reactive behaviour, vigilance, non-consumptive effects, megaherbivore

## Abstract

Animals are faced with a variety of dangers or threats, which are increasing in frequency with ongoing environmental change. While our understanding of fearfulness of such dangers is growing in the context of predation and parasitism risk, the extent to which non-trophic, interspecific dangers elicit fear in animals remains less appreciated. We provide an experimental test for fear responses of savannah ungulates to a dominant and aggressive megaherbivore, the African bush elephant (*Loxodonta africana*), and contrast responses to an apex predator known to elicit fear in this system. Using an automated behavioural response system, we contrast vigilance and run responses of ungulates to elephant, leopard (*Panthera pardus*), and control (red-chested cuckoo *Cuculus solitarius*) vocalizations. Overall, we find that ungulates responded to elephant calls, both in terms of an increase in run and vigilance responses relative to controls. The magnitude of most behavioural responses (four of six considered) to elephant vocalizations were not significantly different than responses to leopards. These results suggest that megaherbivores can elicit strong non-trophic fear responses by ungulates and call to broaden frameworks on fear to consider dominant species, such as megaherbivores, as key modifiers of fear-induced interactions.

## 1. Introduction

Animals live in a dangerous world. When exposed to dangers (e.g. predators, fire), animals often respond through fear-based behavioural responses [1]. Fear is a psychological state that emerges in response to a perceived danger or threat, and it is often quantified based on behaviour, such as fleeing and avoidance [2]. Understanding fear responses by animals to dangers is crucial, as such responses can influence population dynamics, alter species interactions and initiate trophic cascades, thereby reshaping entire communities [3,4]. One common source of danger is trophic interactions. For instance, fear responses to predators are increasingly well understood [5–7], and there is growing recognition of fear responses to parasites [8,9]. Yet if, and to what extent, animals fear other interspecific encounters remains poorly understood [1]. In particular, large, dominant or aggressive species in a community could present non-consumptive threats to species [10–12].

Megaherbivores (mammalian herbivores greater than 1000 kg) are large, dominant, and often aggressive community members that have profound effects on ecological processes that shape ecosystems [13–15]. Despite these well-known effects on ecosystem structure and function [16], the role of megaherbivores on the behaviour of other species remains less appreciated. Some studies suggest megaherbivores can be aggressive to other herbivorous mammals and exclude them from watering holes and foraging sites [17–19]. Yet, it remains unknown if animals generally exhibit fear when they encounter megaherbivores as experiments to understand species fear responses to megaherbivores remain absent.

To determine if megaherbivores can induce fear in other mammals, we contrasted the behavioural responses of medium- to large-sized ungulates to cues of a megaherbivore, the African bush elephant (*Loxodonta africana*), relative to a common predator, the leopard (*Panthera pardus*), in an African savannah. To isolate behavioural responses, we used an automated behavioural response (ABR) system comprised of a motion-activated camera and speaker [20]. This ABR system allowed quantification of reactive responses (as opposed to proactive responses [21]) of ungulates to different cues. Prior research demonstrated that ungulates in our system show strong fear responses to leopards [22]. In our study area, elephant populations have been extirpated for over 100 years, although transient elephants occasionally use the region. While such limited exposure may lead to ungulate naivety of elephant cues, predator recognition by prey from other ecosystems show that some species have an evolved ability to recognize predators and threats which they can maintain for several generations after extirpation [23,24]. Consequently, we predicted that ungulates would be fearful of elephant cues, but that such fear would be weaker than fear for predators. By contrasting responses of elephant cues to the well-understood effects of predators on fear responses by prey, our experimental design provides a benchmark for interpreting the magnitude of potential fear responses of megaherbivores relative to apex predators.

## 2. Methods

### (a) Study area

We conducted this study in low-lying savannahs at three adjacent nature reserves—Mbuluzi Game Reserve (30 km^2^), Mlawula Nature Reserve (165 km^2^) and Hlane Royal National Park (220 km^2^)—that form the majority of the Lubombo conservancy in Eswatini. Dominant overstory trees include marula (*Sclerocarya birrea*) and knobthorn (*Senegalia nigrescens*); dense sicklebush (*Dichrostachys cinerea*) dominates the shrub layer [25]. Common grasses include Guinea grass (*Panicum maximum*) and red grass (*Themeda triandra*). A variety of herbivorous ungulates occur on the reserves, including impala (*Aepyceros melampus*)*,* nyala (*Tragelaphus angasii*), and wildebeest (*Connochaetes taurinus*). Outside of small enclosures (not used in this study), there has not been an extant population of elephants on these reserves in over 100 years.

### (b) ABR system and deployment

To understand the fear response of ungulates to elephants, we used ABRs to play recordings of three vocalizations: elephants, leopards and procedural controls [20,22,26]. The leopard is the primary apex predator in our study area and has previously been shown to induce the greatest fear responses from ungulates in this area [22]. We included leopard vocalizations to contrast with potential elephant responses. For a procedural control, we played vocalizations of the red-chested cuckoo (*Cuculus solitarius*), a common species that vocalizes throughout the day and night in this area.

Between May–July 2022, we placed nine ABRs greater than 600 m apart, further than the daily movements of the common ungulates on our study site [22]. We mounted ABRs following Epperly *et al*. [22]. We set cameras on video, and they began recording once the motion sensor was activated and continued for 20–30 s (night and day, respectively). We set the ABRs to record for 2.5 s before a randomly selected 10 s treatment vocalization was broadcast [22,25]. For treatments, we used 10 different calls (exemplars) for each treatment (for elephants, five rumbles and five trumpets). For more details, see electronic supplementary material, S1.

### (c) Analyses

We scored behaviors before and after the start of the vocalization for each independent video (greater than 60 min between vocalizations of the same species) using Solomon Coder software (Solomon Coder version beta 17.03.22). We focused on two behaviors. First, we determined if the animal(s) ran after the vocalization (see [22,27]). Second, we scored vigilance behaviors using the broad, established categorizations for ungulates of ‘head up’ = vigilance and ‘head down’ = non-vigilance [27,28]. For vigilance responses, we only used observations for ungulates that did not run. For more details on scoring videos, see electronic supplementary material, S1.

We first used a chi-squared test to ensure there were no differences in the proportions of treatments that were randomly applied. Second, to ensure there were not differences in ungulates' pre-treatment behaviour, we compared the proportion of time vigilant prior to vocalizations using a generalized linear mixed model (GLMM) with a beta error distribution in the glmmTMB package [29] in R [30]. For beta GLMMs, we transformed vigilance responses to account for extreme values (y=0 or 1) as: $y^{\prime }=[y(N-1)\;+\;0.5)]/N$, where *y*′ is the transformed response, *y* is the original response, and *N* is the sample size [31]. We considered the proportion of time vigilant before the vocalization as a function of treatment, and we used ABR locations as a random effect. Third, we evaluated run (probability of running) and vigilance (proportion of time vigilant) responses to treatments using similarly parameterized GLMMs for all ungulates combined and separately for individual species with at least 15 observations per treatment (electronic supplementary material, tables S1, S2). For each model, we used the emmeans package [32] to calculate estimates and SEs and to conduct pairwise comparisons among treatments with Tukey's adjustment term. In one test, complete separation of run response data occurred because 0 individuals ran to control playbacks. In this case, we used the blme package [33] to fit a similar model but imposing vague normal priors (mean = 0, s.d. = 3) on treatment effects. Finally, we re-ran models that decomposed the elephant treatments into the two call types, rumble versus trumpet, to assess if and how elephant call type might influence fear responses, as trumpet calls are likely more aggressive vocalizations than rumbles [34].

## 3. Results

**Figure 1.**
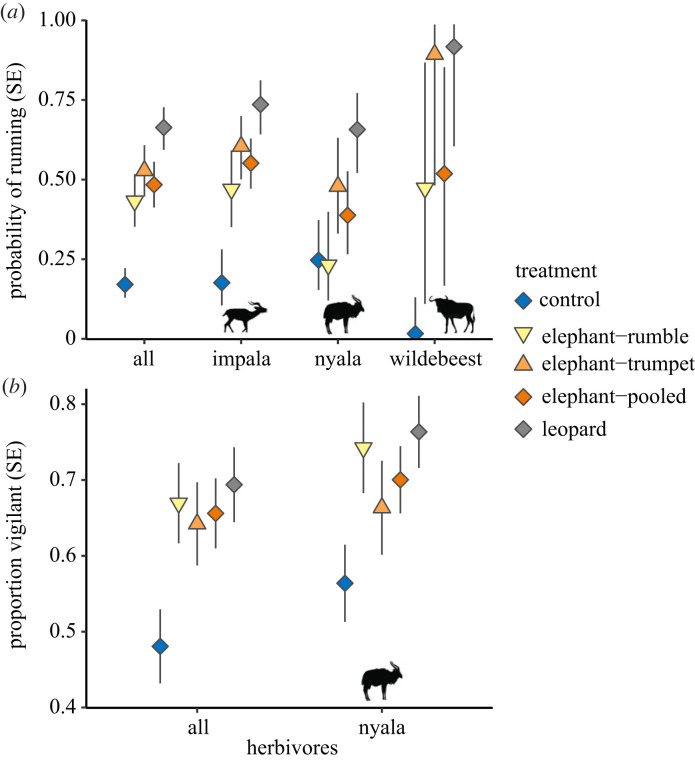
Fear responses of ungulates to treatments. (*a*) The probability of running and (*b*) the proportion of time spent vigilant (SEs) by ungulates in response to elephant, leopard and control (red-chested cuckoo) vocalizations. In both, shown are responses of all ungulate herbivores combined and responses for ungulates with at least 15 observations per treatment. For elephant treatments, we decompose vocalization playbacks (elephant–pooled) into responses to elephant rumbles and trumpets.

We recorded a total of 483 scorable independent videos from 10 target species (electronic supplementary material, tables S1, S2). Combined across species, there was a strong overall effect of treatments ($\chi^{2}=61.1$, *p* < 0.0001), where individuals ran significantly more frequently to both elephant and leopard vocalizations compared to control (bird) vocalizations (electronic supplementary material, table S3). The magnitudes of effects were large: running was 2.8× more frequent to elephant vocalizations and 3.9× more frequent to leopard vocalizations than to controls (figure 1*a*). Animals also ran significantly more (40%) to leopard than to elephant vocalizations (figure 1*a*, electronic supplementary material, table S3). For the animals that did not run, there was a strong overall effect of treatments on vigilance ($\chi^{2}=29.06$, *p* < 0.0001), where they significantly increased vigilance after the vocalizations of elephants (*β* = 0.68, *p* = 0.0001) and leopards (*β* = 0.88, *p* < 0.0001) relative to controls. However, there was no difference between the amount of post-treatment vigilance between elephant and leopard treatments (*β* = −0.20, *p* = 0.50; figure 1*b*, electronic supplementary material, table S4). We found no difference in vigilance among treatments ($\chi^{2}=0.45$, *p* = 0.80) in the pre-treatment behaviour for the 2.5 s prior to vocalizations. When pooling across species, run and vigilance responses to both trumpet and rumble calls of elephants were greater than controls and there was no significant difference in responses to elephant trumpets versus rumbles (*p* > 0.6; electronic supplementary material, table S5, S6, figure S1). Yet there was a slight tendency for trumpet calls to elicit more run responses than rumbles: response to rumbles was less than leopards (*p* = 0.007) but response to trumpets was not (*p* = 0.159).

We found variation in the response of the most commonly detected species, impala (*n* = 118), nyala (*n* = 144), and wildebeest (*n* = 65). Wildebeest and impala showed a similar pattern: both ran more in response to elephant (wildebeest = 3.8× more; impala = 3.1× more) and leopard (wildebeest = 4.8× more; impala = 4.5× more) vocalizations than to controls (figure 1*a*). Yet, we found no significant differences between run responses to elephant (rumbles and trumpets pooled) and leopard vocalizations (electronic supplementary material, table S3). In contrast, nyala ran more from leopard vocalizations than both elephant and control vocalizations and we found no difference between nyala run response to elephant and control vocalizations (figure 1*a*, electronic supplementary material, table S3). For vigilance responses, we had sufficient sample size only for nyala to test effects of treatments. There was an overall effect of treatments on vigilance ($\chi^{2}=8.39$, *p* = 0.015); nyala significantly increased vigilance to elephant (*β* = 0.51, *p* = 0.041) and leopard (*β* = 0.77, *p* < 0.008) treatments relative to controls. There was no significant difference in nyala vigilance to elephants and leopards (electronic supplementary material, table S4). When comparing elephant rumble and trumpet calls at the species level, there were no significant differences in responses to call type (*p* > 0.4; electronic supplementary material, table S5, S6). Relative to controls, run responses to rumble calls were only marginally significant for impala (*p* = 0.1). Both impala and wildebeest significantly increased run responses to elephant trumpets (figure 1). Other comparisons showed no significant effect relative to controls (electronic supplementary material table S5, S6).

## 4. Discussion

We revealed strong fear responses of ungulates to elephant vocalizations. The effects on individuals running from elephant vocalizations were slightly weaker on average compared to the most lethal predator on our site (leopard) but the amount of vigilance behaviour was comparable between elephant and leopard vocalizations. Moreover, our average ungulate run response (48%) to elephant vocalizations was comparable to their response to wild dogs (*Lycaon pictus*) (47%) [35] and greater than their response to spotted hyena (*Crocuta crocuta*) (35%) and domestic dog (*Canis familiaris*) vocalizations (34%) quantified in a study using a similar approach in this area [22]. However, this run response varied in magnitude across ungulate species from 74% of impala running compared with only 39% of nyala (figure 1*a*). Across all comparisons, four of six primary responses revealed no significant differences in response to elephants and leopards, suggesting that elephants are generally perceived as dangerous to ungulate herbivores and call to broaden the frameworks on fear to consider dominant species, such as megaherbivores, as key interactions.

The responses of ungulates to elephant vocalizations could be driven by at least three mechanisms. First, resource competition could drive responses, such that species that overlap in resource use may have greater fear responses than other species [36]. There was some support for this hypothesis with impala showing the strongest response to elephants and having the more comparable diets to elephants than other species we considered [37] and habitat use overlaps with elephants [38,39]. Yet nyala also share habitat preferences with elephants [39], and elsewhere have been shown to run less frequently but be more vigilant to predator playbacks than impala in this system [22], suggesting that differences in responses by impala and nyala may be driven by different types of anti-predator behavioural strategies. Second, responses could be due to aggressive behaviors by elephants to other species. Observations from water holes suggest that elephants may be aggressive to other species in such situations [18,19], yet it remains unclear whether elephants vary in their aggression to different species and if aggressive behaviors near water holes transfer to fear by ungulates in other areas. Trumpeting by elephants can occur in response to fear and can represent a more aggressive response than rumbles (which are used in social activities) [34]; we found no significant differences in ungulate responses to these different call types, although trumpeting tended to elicit slightly more frequent run responses than rumbles and more often elicited responses that were similar to responses to leopards (figure 1; electronic supplementary material, table S5, S6). While sample sizes were more limited for interpreting elephant call types, these results suggest that certain animals may have learned to elude elephants that show signs of aggressive behaviors by running, while being generally more vigilant in the presence of elephants otherwise. Finally, responses to elephants could be based on generalized responses to similar cues or generalized neophobia. In this way, ‘fear generalization’ could be an imperfect but largely successful strategy to danger [1,40]. Because elephants were extirpated in this system over 100 years ago and only transient elephants have occurred since, we expect that these responses reflect either innate responses or imperfect generalized responses based on other megaherbivores in this region (e.g. *Hippopotamus amphibius*). Experiments designed to test these alternative hypotheses could help shed light on why fear of megaherbivores may arise.

Regardless of the mechanism behind these responses, these results suggest that fear responses can emerge not just from predators, but from megafauna such as elephants more broadly, which has several potential consequences. First, these results point to the potential of cascading effects of elephants on the behaviour of other species in savanna ecosystems. While the direct effects of elephants on ecosystem structure and function are increasingly understood [16], elephants may also have indirect effects on savanna ecosystems by generating changes in the behaviour of ungulates. Second, our results suggest that in ecosystems where elephants have been lost, rewilding of elephants [41–43] may benefit from ungulates retaining their fear responses, as observed in our study.

Our experiment examined reactive responses of ungulates to elephant cues but was not designed to understand proactive responses [21]. Other observations focused on proactive responses in ungulate species suggests that dominant species may not elicit strong interspecific avoidance [36]. However, interspecific aggression appears to be common across the vertebrate tree of life [11,44], highlighting the need for experiments aimed at understanding potential effects. Wells *et al*. [45] provided experimental evidence that megaherbivores may elicit changes in habitat use of other ungulates, where several species avoided areas where megaherbivores were present. Our results suggest that patterns like these may be driven by fear and call for a better understanding of how megaherbivores may alter the behaviour of other species and its consequences for communities and ecosystems.

## Data Availability

Data and code to reproduce the analyses provided in the electronic supplementary material [46].
